# Operation for Removal of Malignant Epulis

**Published:** 1861-08

**Authors:** 


### The Clinique.

### MERCY HOSPITAL—SURGICAL WARDS.

Service of Prof. E. ANDREWS, M. D., Attending Surgeon.

Reported for the EXAMINER.

GENTLEMEN:—The patient before us presents a well developed example of the tumor called *Epulis.* This term, which has come down to us on the venerable authority of the Greek physicians, is derived from two words, which signify, "upon the gum": but with all due respect to the shades of old surgical heroes, the name is an unfortunate one, for the tumor is not *upon* the gum, but *under* it. However, let us not insult the dead, for a glance at the most modern surgical works will show you that our best writers have not, in this disease, advanced a step beyond Hippocrates.

The term epulis is loosely applied to all kinds of tumors springing from the alveolar processes. As some of them are benign and others malignant, and as each writer describes the kind which has most attracted his own attention, we have given us the most contradictory accounts of this disease. The truth is, the word epulis should be abandoned, because it only indicates the locality of the disease, and we should speak simply of a fibrous, a bony, a benign or a malignant tumor of the alveolar processes, just as we do in any other part of the body.

The case before us is of the malignant variety. It has been in existence about six years, but until the past two months, its growth was slow. During the latter period, it has taken on a great activity, and has now attained a size which will necessitate a loss of a portion of the lower maxilla. At first, these cases present a small, innocent looking tumor at the roots of some of the teeth, elevating the gum, and having a more or less constricted base. For a long time it may occasion no remarkable inconvenience, but at length it will be observed that one or more of the teeth are loosened, and that the adjoining ones are gradually falling into the same condition. Finally, they drop out, and an open sore is the result. The disease continues to spread, contaminating the glands of the neck and destroying the organs of the mouth, until the patient sinks and dies. In the present case, the growth has loosened all the incisor teeth of the lower jaw, and projects both on the inner and outer side of the symphysis. Also, you observe, there is a hard, scirrhus-like lymphatic gland under the right side of the jaw, and connected to the present tumor.

The malignant epulis is generally of the variety called cancroid, and like cancroid diseases of the lip, it is capable of cure by early excision. In all cases, however, it must be remembered that the tumor springs from the bone itself, and hence the excision must remove all the affected portion of the alveolar process,—otherwise it will certainly return. A mere shaving off of the soft parts will be of no use whatever. The best method is usually to administer an anæsthetic, and then proceed to extract all the loosened teeth. Next, to make sure work, remove one firm tooth on either side of the gap. Carry an incision around the tumor, at a little distance from it, cutting quite down to the bone, and remove the growth by dissection. If nearly the whole thickness of the jaw is affected, a section of it may be sawn out, but usually it will be sufficient to cut away the affected alveolar processes, with the gouge forceps, continuing the removal until you reach perfectly sound bone.

In the present instance, the patient has neglected herself rather too long, and I fear for the ultimate result, but yet I consider an operation to be her only hope.

As she is now sufficiently under the influence of the ether, I will commence by extracting the loosened incisors. You observe that the canines are firm, but it will be safest to extract them also, because it is almost certain that their sockets on the side next to the incisors, are more or less contaminated. This being accomplished, and the soft part of the tumor cut away, I will cut down with the saw on either side, about two-thirds the depth of the jaw, and remove the intervening bone with gouge forceps. Thus you see we have excised every visible trace of the original disease. There only remains the hard tumor under the right side of the jaw. This, from its situation, must be taken out by an incision through the skin. Carrying my scalpel, therefore, along the base of the maxilla, you observe that I cut through the skin, fasciæ, and the thin plane of the platysma myoides muscle. The tumor is then easily dissected out, except the line of connection with the original epulis. This indurated chord I isolate, by pushing a straight bistoury through into the floor of the mouth, and carrying it around the offending tissue.

Thus we have removed the whole of the disease, so far as we can judge, and as the malignancy of this form of epulis is a modified form, there is a reasonable hope of permanent cure. I confess to you, however, I would much rather that this case had come to me earlier, before any progress had been made beyond the alveolar process. As it is, we have done our duty, and given the patient the best chance which the present advanced stage of the disease admits.

